# Effects of novel specialty soy protein products on growth performance and fecal dry matter of nursery pigs

**DOI:** 10.1093/tas/txag066

**Published:** 2026-05-16

**Authors:** Jessica L Smallfield, Mike D Tokach, Jason C Woodworth, Joel M DeRouchey, Katelyn N Gaffield, Robert D Goodband, Jordan T Gebhardt, Alan J Warner, Chad W Hastad, Long Zou, Sabrina B May, Wesley P Schweer, Chad M Pilcher

**Affiliations:** Department of Animal Sciences and Industry, College of Agriculture, Kansas State University, Manhattan, KS, 66506-0201, United States; Department of Animal Sciences and Industry, College of Agriculture, Kansas State University, Manhattan, KS, 66506-0201, United States; Department of Animal Sciences and Industry, College of Agriculture, Kansas State University, Manhattan, KS, 66506-0201, United States; Department of Animal Sciences and Industry, College of Agriculture, Kansas State University, Manhattan, KS, 66506-0201, United States; Department of Animal Sciences and Industry, College of Agriculture, Kansas State University, Manhattan, KS, 66506-0201, United States; Department of Animal Sciences and Industry, College of Agriculture, Kansas State University, Manhattan, KS, 66506-0201, United States; Department of Diagnostic Medicine/Pathobiology, College of Veterinary Medicine, Kansas State University, Manhattan, KS, 66506-0201, United States; New Fashion Pork, Jackson, MN, United States; New Fashion Pork, Jackson, MN, United States; Bunge North America Inc, Chesterfield, MO, United States; Cargill Animal Nutrition, Wayzata, MN, United States; Cargill Animal Nutrition, Wayzata, MN, United States; Cargill Animal Nutrition, Wayzata, MN, United States

**Keywords:** fecal dry matter, nursery pig, soy protein concentrate, specialty soy protein, thermo-mechanically processed soybean meal

## Abstract

Two experiments were conducted evaluating the effects of specialty soy protein products on nursery pig growth performance and fecal dry matter (DM). In Exp. 1, 360 barrows (DNA 200 × 400; initially 5.6 ± 0.02 kg) were used in a 37-d study to assess a refined soy protein concentrate (SPC) as a replacement for soybean meal (SBM) on a standardized ileal digestible (SID) Lys basis. Diets contained increasing SPC (0, 4.25, 8.5, 12.75, and 17%) replacing 0, 25, 50, 75, or 100% of SBM, and a positive control containing 8.5% enzymatically treated SBM (ESBM). Average daily gain (ADG) and gain-to-feed ratio (G:F) for the experimental period (d 0 to 23) increased (quadratic, *P* < 0.05) then decreased as SPC increased, with the greatest improvement observed when SPC replaced up to 50% of the SBM. Additionally, average daily feed intake (ADFI) increased (linear, *P* = 0.019) as SPC increased. Overall (d 0 to 37), increasing SPC increased (quadratic, *P* = 0.014) ADG and tended to increase (quadratic, *P* = 0.080) ADFI with the greatest response at 50% and 75% replacement of SBM, respectively. Gain-to-feed ratio tended to decrease (linear, *P* = 0.087) in pigs previously fed increasing SPC for the first 23 d. Fecal DM increased as SPC increased (linear, *P* = 0.035) on d 9, and pigs fed 50% SBM replacement (8.5% SPC) had greater (*P* = 0.011) fecal DM than those fed 8.5% ESBM. In Exp. 2, 1254 mixed-sex pigs (PIC 800 × [Fast LW × PIC L02]; initially 5.6 ± 0.10 kg) were used in a 28-d study to evaluate thermo-mechanically processed SBM (TM-SBM) at increasing levels replacing SBM on a SID Lys basis (0, 25, 50, 75, and 100% in phase 1 and 0, 12.5, 25, 37.5, and 50% in phase 2). For the experimental period (d 0 to 21), ADG and ADFI increased then decreased (quadratic, *P* ≤ 0.038) with the greatest improvement observed when TM-SBM replaced 25% to 50% of the SBM from d 0 to 7 and 12.5% to 25% from d 7 to 21 with an increase in G:F (quadratic, *P* = 0.036) observed with TM-SBM replacing 50% and 25% of the SBM from d 0 to 7 and d 7 to 21, respectively, but worsening thereafter. A treatment × day interaction was observed for fecal DM (quadratic, *P* = 0.024). On d 7, fecal DM increased when TM-SBM replaced 25% of the SBM (quadratic, *P* = 0.004), but decreased with 50% replacement before remaining constant, with no differences on d 21. Ultimately, replacing 25% to 50% of the digestible Lys provided by SBM in diets for 5- to 13-kg pigs with specialty soy products improved growth performance and fecal DM under the dietary formulation conditions used in these experiments.

## Teaser

Replacing 25% to 50% of the digestible lysine provided by soybean meal in nursery pig diets with the specialty soy products tested improved growth performance.

## Introduction

Weaning is a stressful period for pigs where they experience physiological, environmental, and social changes (Campbell et al. 2013). One of the most significant challenges is the sudden change in diet from a highly digestible, liquid milk-based diet to a solid, plant-based diet usually comprised of corn and soybean meal (SBM). Soybean meal is an economical protein source for use in swine diets, but it needs to be gradually introduced into diets of weanling pigs. This is due to the presence of complex carbohydrates, like stachyose and raffinose, trypsin inhibitors, and antigens, such as glycinin and β-conglycinin (Zheng et al. 2021). These components present at a high level can cause hypersensitivity in nursery pigs (Hao et al. 2009), which may negatively affect growth performance and increase the risk of post-weaning diarrhea. Weanling pigs also experience low feed intake, which worsens post-weaning challenges (Le Dividich and Sève 2000). Therefore, incorporating protein sources with lower anti-nutritional factors (ANF) and higher digestibility is important to encourage feed intake in weaned pigs.

Further processing of soy products can improve digestibility and tolerance in weanling pigs compared to conventional SBM (Pedersen et al. 2016). However, specialty soy products can vary in nutrient profile, levels of complex carbohydrates, ANF, and palatability. Several refined SBM-based products have been developed to mitigate limitations such as ANF in nursery pig diets, including enzymatically treated SBM (Long et al. 2021), soy protein concentrate (Deng et al. 2022), fermented SBM (Yuan et al. 2017), and other specialty soy proteins. Each processing method affects protein quality, digestibility, palatability, and nutritional composition differently.

Soy protein concentrate is produced by removing soluble carbohydrates and other non-protein components from defatted soy flakes through ethanol extraction, resulting in a product that contains approximately 65% protein (Peisker 2001). Research using soy protein concentrates has shown variable results, with some reporting a positive effect on growth performance when replacing animal protein supplements (Deng et al. 2023), while others have reported negative effects on growth performance compared to animal protein products (Nofrarías et al. 2007; Che et al. 2012). A specific refined soy protein concentrate (SPC; PurePro Soy, Bunge North America Inc., Chesterfield, MO) that is commercially available for use in diets has reduced levels of complex carbohydrates like stachyose (1.0%) and raffinose (0.1%). Whereas conventional SBM can contain approximately 5.2% stachyose and 1.1% raffinose (Cervantes-Pahm and Stein 2010). Additionally, it has lower concentrations of glycinin and β-conglycinin (less than 3% and 2%, respectively) compared to conventional SBM which can have concentrations of approximately 14.3% glycinin and 10.1% β-conglycinin (Park et al. 2020). With reduced ANF, increased quantities of specialty processed soy protein may be included in weanling pig diets as replacement for other specialty protein sources.

Another further processed specialty soy protein product has recently become available in the United States and is produced by thermo-mechanical processing of SBM (TM-SBM; Provisoy, Cargill Animal Nutrition, Wayzata, MN). This additional processing step reduces ANF, such as trypsin inhibitors, compared to conventional SBM. This specific thermo-mechanically processed SBM has been shown to support gut health (Hu et al. 2023a) and improve protein digestibility (Hu et al. 2023b) in *Escherichia coli* challenged pigs. However, no peer-reviewed published data exist for this product when evaluated in a non-challenged, commercial setting in the United States.

Because specialty soy products differ in crude protein concentration and amino acid profile relative to SBM, replacement on a SID Lys basis may alter dietary nitrogen supply at high inclusion levels. Limited data is available that describes the optimum dietary addition of these specialty protein sources in diets for newly weaned pigs. Therefore, the objective of these two experiments was to evaluate the effects of replacing SBM with specialty soy proteins on nursery pig growth performance and fecal dry matter. It was hypothesized that partially replacing SBM with specialty soy products would improve nursery pig growth performance and increase fecal dry matter.

## Materials and methods

The Kansas State University Institutional Animal Care and Use Committee approved the protocols used in these experiments (IACUC #4506.19 and #4892). Nutrient profiles for the specialty soy protein products tested were obtained from the manufacturers for diet formulation. Samples of the products were analyzed at the University of Missouri Agricultural Experiment Station Chemical Laboratory for CP (Method 990.03; AOAC International, 2006), ash, dry matter (DM; Method 935.29; AOAC International, 2006), ether extract (ANKOM Technology, 2004), crude fiber (ANKOM Technology, 2005), and complete AA profile (Method 982.30; AOAC International, 2006; Table 1).

**Table 1.** Analyzed composition of specialty soy protein sources for Exp. 1 and 2 (as-fed basis)^a^

	Specialty soy protein	
*Nutrient, %*	Soy protein concentrate (SPC)^b^	Thermo-mechanically processed soybean meal (TM-SBM)^c^
* CP*	66.98	48.57
* Dry matter*	93.12	93.30
* Crude fat*	0.14	1.43
* Crude fiber*	3.94	3.92
* Ash*	5.42	6.81
* Essential AA*		
* Arg*	4.82	3.46
* His*	1.78	1.27
* Ile*	3.34	2.38
* Leu*	5.30	3.73
* Lys*	4.34	3.13
* Met*	0.91	0.67
* Phe*	3.46	2.50
* Thr*	2.56	1.86
* Trp*	0.94	0.68
* Val*	3.50	2.47
* Non-essential AA*		
* Ala*	2.91	2.10
* Asp*	7.43	5.36
* Cys*	0.94	0.76
* Glu*	12.58	8.88
* Gly*	2.82	2.05
* Pro*	3.48	2.49
* Ser*	2.73	2.00
* Tyr*	2.36	1.83

a Samples were analyzed for proximate analysis and complete AA profile (University of Missouri Agricultural Experiment Station Chemical Laboratory).

b PurePro Soy; Bunge; Chesterfield, MO.

c Provisoy; Cargill Animal Nutrition; Wayzata, MN.

### Experiment 1

Experiment 1 was conducted at the Kansas State University Segregated Early Weaning facility in Manhattan, KS. The facility has two identical barns that are completely enclosed, environmentally controlled, and mechanically ventilated. Each pen contained a 4-hole, dry self-feeder and a cup waterer for ad libitum access to feed and water. Pens (1.2 × 1.2 m) had metal tri-bar floors and allowed approximately 0.25 m^2^/pig.

A total of 360 barrows (DNA 200 × 400; initially 5.6 ± 0.02 kg) were used in a 37-d growth trial. Pigs were weaned at approximately 21 d of age and blocked by initial weight. Pens were assigned in a generalized randomized block design to one of six dietary treatments. Pigs were blocked into light (initially 5.0 ± 0.01 kg) and heavy groups (initially 6.2 ± 0.01 kg). There were five pigs per pen, and within each block, there were six pens per treatment (three pens per weight group in each barn) for a total of 12 replications per treatment. Diets were corn-soybean meal-based and consisted of increasing SPC (0, 4.25, 8.5, 12.75, and 17%) replacing 0, 25, 50, 75, or 100% of the standardized ileal digestible (SID) Lys provided by SBM in the diet, respectively. A sixth diet served as a positive control containing 8.5% enzymatically treated SBM (HP 300, Hamlet Protein; Findlay, OH) replacing a portion of the SBM in the diet (Tables 2 and 3). All diets were formulated to the same SID Lys and net energy (NE) levels, with SBM net energy (NE) considered to be 92% of corn NE. However, diets were not balanced for CP concentration, which reflects practical formulation approaches but resulted in increasing SID Lys:CP ratio as SPC replaced SBM. Dietary additions of feed-grade amino acids (AA) were adjusted to meet or exceed AA requirements in relation to Lys for Ile, Met and Cys, Thr, Trp, and Val. Pigs were fed treatment diets in two phases with phase 1 from d 0 to 9, followed by phase 2 from d 9 to 23. Subsequently, all pigs were fed a common corn-soybean meal-based diet without specialty soy protein sources until d 37 of the trial.

**Table 2.** Ingredient composition of **Exp**. 1 diets (as-fed basis)^a^

	Phase 1					
	SPC replacement of SBM, %					ESBM, %
	0	25	50	75	100	8.50
*Ingredient, %*						
* Corn*	43.02	46.20	49.41	52.63	55.88	44.55
* Soybean meal (47% CP)*	30.00	22.51	15.02	7.52	—	19.98
* Soy protein concentrate^b^*	—	4.25	8.50	12.75	17.00	—
* Enzymatically treated SBM^c^*	—	—	—	—	—	8.50
* Whey powder*	22.50	22.50	22.50	22.50	22.50	22.50
* Soybean oil*	1.00	1.00	1.00	1.00	1.00	1.00
* Calcium carbonate*	0.65	0.65	0.65	0.65	0.65	0.65
* Monocalcium P (21% P)*	0.68	0.73	0.75	0.78	0.80	0.70
* Salt*	0.30	0.30	0.30	0.30	0.30	0.30
* L-Lys-HCl*	0.39	0.39	0.39	0.39	0.39	0.39
* DL-Met*	0.22	0.22	0.22	0.22	0.22	0.22
* L-Thr*	0.18	0.19	0.19	0.20	0.20	0.18
* L-Trp*	0.03	0.04	0.05	0.05	0.06	0.03
* L-Val*	0.12	0.12	0.11	0.11	0.10	0.09
* Zinc oxide*	0.39	0.39	0.39	0.39	0.39	0.39
* Vitamin premix*	0.25	0.25	0.25	0.25	0.25	0.25
* Trace mineral premix*	0.15	0.15	0.15	0.15	0.15	0.15
* Choline chloride*	0.05	0.05	0.05	0.05	0.05	0.05
* Phytase^d^*	0.08	0.08	0.08	0.08	0.08	0.08
* Total*	100	100	100	100	100	100
*Calculated analysis*						
*SID AA, %*						
* Lys, %*	1.35	1.35	1.35	1.35	1.35	1.35
* Ile:Lys*	59	59	59	59	59	61
* Leu:Lys*	114	115	116	117	117	116
* Met:Lys*	37	37	37	37	38	36
* Met and Cys:Lys*	58	58	58	58	58	58
* Thr:Lys*	65	65	65	65	65	65
* Trp:Lys*	20.0	20.1	20.2	20.2	20.1	20.0
* Val:Lys*	70	70	70	70	70	70
* His:Lys*	35	34	34	33	33	35
*NE, kcal/kg*	2,606	2,604	2,604	2,604	2,604	2,590
*SID Lys:NE, g/Mcal*	5.18	5.18	5.18	5.18	5.18	5.21
*Lactose, %*	16.2	16.2	16.2	16.2	16.2	16.2
*SID Lys:CP*	6.47	6.58	6.69	6.81	6.93	6.42
*CP, %*	20.8	20.5	20.2	19.8	19.4	21.0
*Ca, %*	0.69	0.69	0.69	0.69	0.68	0.69
*STTD P, %*	0.55	0.55	0.55	0.55	0.55	0.56
*Ca:P*	1.11	1.11	1.11	1.11	1.12	1.11

a Phase 1 diets were fed from d 0 to 9 (5.6 to 6.8 kg).

b SPC; soy protein concentrate; PurePro Soy (Bunge, Chesterfield, MO).

c ESBM; enzymatically treated soybean meal; HP 300 (Hamlet Protein, Findlay, OH).

d Ronozyme HiPhos (DSM, Parsippany, NJ) included at 2,027 FTU/kg provided an estimated release of 0.14% STTD P.

**Table 3.** Ingredient composition of Exp. 1 diets (as-fed basis)^a^

	Phase 2						Phase 3
	SPC replacement of SBM, %					ESBM, %	
	0	25	50	75	100	8.50	Common
*Ingredient, %*							
* Corn*	56.13	59.32	62.53	65.74	68.95	57.61	68.06
* Soybean meal (47% CP)*	29.99	22.50	15.00	7.51	—	20.01	28.12
* Soy protein concentrate^b^*	—	4.25	8.50	12.75	17.00	—	—
* Enzymatically treated SBM^c^*	—	—	—	—	—	8.50	—
* Whey powder*	10.00	10.00	10.00	10.00	10.00	10.00	—
* Calcium carbonate*	0.70	0.70	0.70	0.70	0.70	0.70	0.75
* Monocalcium P (21% P)*	0.75	0.80	0.83	0.85	0.88	0.75	0.85
* Salt*	0.55	0.55	0.55	0.55	0.55	0.55	0.60
* L-Lys-HCl*	0.50	0.50	0.50	0.50	0.50	0.50	0.55
* DL-Met*	0.23	0.23	0.23	0.23	0.23	0.23	0.21
* L-Thr*	0.24	0.24	0.24	0.25	0.25	0.24	0.23
* L-Trp*	0.05	0.06	0.07	0.07	0.08	0.05	0.05
* L-Val*	0.15	0.15	0.15	0.14	0.14	0.15	0.16
* Zinc oxide*	0.25	0.25	0.25	0.25	0.25	0.25	—
* Vitamin premix*	0.25	0.25	0.25	0.25	0.25	0.25	0.25
* Trace mineral premix*	0.15	0.15	0.15	0.15	0.15	0.15	0.15
* Phytase^d^*	0.08	0.08	0.08	0.08	0.08	0.08	0.03
* Total*	100	100	100	100	100	100	100
*Calculated analysis*							
*SID AA, %*							
* Lys, %*	1.35	1.35	1.35	1.35	1.35	1.35	1.30
* Ile:Lys*	55	55	55	55	55	57	53
* Leu:Lys*	112	113	114	115	115	114	113
* Met:Lys*	37	37	38	38	38	37	36
* Met and Cys:Lys*	58	58	58	58	58	58	57
* Thr:Lys*	65	65	65	65	65	65	63
* Trp:Lys*	20.1	20.0	20.3	20.3	20.2	20.1	19.3
* Val:Lys*	70	70	70	70	70	72	70
* His:Lys*	35	34	34	34	33	35	35
*NE, kcal/kg*	2,549	2,549	2,549	2,549	2,549	2,537	2,449
*SID Lys:NE, g/Mcal*	5.29	5.29	5.29	5.29	5.29	5.33	5.31
*Lactose, %*	7.2	7.2	7.2	7.2	7.2	7.2	—
*SID Lys:CP*	6.53	6.64	6.76	6.88	7.00	6.47	6.50
*CP, %*	20.6	20.3	20.0	19.6	19.3	20.9	20.0
*Ca, %*	0.65	0.65	0.65	0.65	0.64	0.65	0.61
*STTD P, %*	0.50	0.50	0.50	0.50	0.50	0.50	0.43
*Ca:P*	1.10	1.10	1.11	1.11	1.12	1.10	1.08

a Phase 2 was fed from d 9 to 23 (6.8 to 12.7 kg). Phase 3 was fed from d 23 to 37 (12.7 to 20.4 kg).

b SPC; soy protein concentrate; PurePro Soy (Bunge, Chesterfield, MO).

c ESBM; enzymatically treated soybean meal; HP 300 (Hamlet Protein, Findlay, OH).

d Ronozyme HiPhos (DSM, Parsippany, NJ) included at 2,027 FTU/kg provided an estimated release of 0.14% STTD P.

Treatment diets were manufactured at the Kansas State University O.H. Kruse Feed Technology Innovation Center in Manhattan, KS and fed in pellet form for phase 1 and meal form in phases 2 and 3. Pig weights and feed disappearance were measured on d 0, 9, 16, 23, 30, and 37 to determine average daily gain (ADG), average daily feed intake (ADFI), and gain-to-feed ratio (G:F). Feces were collected at the end of phases 1 (d 9) and 2 (d 23) from the same three randomly selected pigs in each pen for dry matter (DM) analysis. Fecal samples were dried at 55°C in a forced air oven for 48 h, and the ratio of dried to wet fecal weight determined the percentage fecal dry matter. Fecal samples were analyzed separately for each pig, and the average of the three samples from each pen was then used for statistical analysis.

### Experiment 2

Experiment 2 was conducted at the New Fashion Pork (Jackson, MN) research wean-to-finish facility to evaluate the effect of a specialty TM-SBM in a commercial research setting. The facility consisted of two rooms with a total of 62 pens. The facility was completely enclosed, environmentally regulated, and mechanically ventilated. Each pen contained a three-hole, dry self-feeder and a bowl waterer for ad libitum access to feed and water.

A total of 1,254 mixed-sex pigs (PIC 800 × [Fast LW × PIC L02]; initially 5.6 ± 0.10 kg) were used in a 28-d growth study. Pens of pigs were randomly allotted to one of five dietary treatments in a randomized complete block design with initial body weight (BW) and nursery entry date as blocking factors. There were 18 to 20 pigs per pen and 12 pens per treatment. The experimental diets were corn-soybean meal-based with increasing TM-SBM (0, 5.79, 11.58, 17.36, and 23.02%) replacing 0, 25, 50, 75, and 100% of the SID Lys provided by SBM in phase 1 and 0, 3.21, 6.41, 9.62, and 12.82% replacing 0, 12.5, 25, 37.5, and 50% in phase 2 (Table 4). All diets were formulated to the same SID Lys level. Similar to Exp. 1, CP was allowed to decrease with increasing TM-SBM inclusion, resulting in increasing SID Lys: CP ratio at higher replacement levels. The same amount of L-Lys HCl was used in each diet within phase, but additions of other feed-grade AA were adjusted to equalize Met and Cys, Thr, Trp, and Val concentrations relative to Lys in all diets. Nutrient loading values for TM-SBM were provided by the supplier and values for the other ingredients were obtained from the NRC (2012). Like Exp. 1, treatment diets were fed in two phases but with a feed budget of 2.7 kg per pig for phase 1 (d 0 to 7) and 4.5 kg for phase 2 (d 7 to 21) followed by a common diet from d 21 to 28 (phase 3).

**Table 4.** Ingredient composition of Exp. 2 diets (as-fed basis)^a^

	Phase 1					Phase 2					Phase 3
*TM-SBM replacement of SBM, %:*	0	25	50	75	100	0	12.5	25	37.5	50	Common
*Ingredient, %*											
* Corn*	51.27	51.70	52.09	52.49	52.88	57.80	58.02	58.25	58.48	58.71	65.56
* Soybean meal (47% CP)*	24.58	18.40	12.22	6.04	—	27.24	23.82	20.40	16.97	13.55	30.43
* Thermo-mechanically processed SBM* ^b^	—	5.79	11.58	17.36	23.02	—	3.21	6.41	9.62	12.82	—
* Spray-dried whey*	16.65	16.65	16.65	16.65	16.65	10.00	10.00	10.00	10.00	10.00	—
* Bovine blood plasma*	2.00	2.00	2.00	2.00	2.00	—	—	—	—	—	—
* Choice white grease*	2.00	2.00	2.00	2.00	2.00	1.00	1.00	1.00	1.00	1.00	—
* Calcium carbonate*	0.45	0.45	0.44	0.43	0.43	0.69	0.68	0.68	0.67	0.66	0.68
* Monocalcium P (21% P)*	0.93	0.90	0.89	0.87	0.85	0.92	0.92	0.90	0.89	0.88	1.00
* Salt*	0.40	0.40	0.41	0.42	0.43	0.55	0.56	0.56	0.57	0.57	0.60
* L-Lys-HCl*	0.38	0.38	0.38	0.38	0.38	0.50	0.50	0.50	0.50	0.50	0.50
* DL-Met*	0.20	0.21	0.21	0.22	0.22	0.22	0.22	0.23	0.23	0.23	0.28
* L-Thr*	0.18	0.18	0.18	0.19	0.19	0.24	0.24	0.24	0.24	0.24	0.25
* L-Trp*	0.03	0.03	0.04	0.04	0.05	0.05	0.05	0.06	0.06	0.06	0.05
* L-Val*	0.10	0.09	0.09	0.08	0.07	0.15	0.15	0.14	0.13	0.13	0.20
* L-Ile*	—	—	—	—	—	—	—	—	—	—	0.03
* Zinc oxide*	0.40	0.40	0.40	0.40	0.40	0.26	0.26	0.26	0.26	0.26	—
* Vitamin premix with phytase* ^c^	0.25	0.25	0.25	0.25	0.25	0.25	0.25	0.25	0.25	0.25	0.25
* Trace mineral premix*	0.15	0.15	0.15	0.15	0.15	0.15	0.15	0.15	0.15	0.15	0.15
* Choline chloride*	0.05	0.05	0.05	0.05	0.05	—	—	—	—	—	—
* Total*	100	100	100	100	100	100	100	100	100	100	100

a Phase 1 diets were fed from d 0 to 7 (2.7 kg feed budget). Phase 2 diets were fed from d 7 to 21 (4.5 kg feed budget). Phase 3 was fed to pigs from d 21 to 28.

b TM-SBM; thermo-mechanically processed soybean meal; Provisoy (Cargill Animal Nutrition, Minneapolis, MN).

c Empirical Phytase (ADM, Chicago, IL) included at 3,132 FTU/kg provided an estimated release of 0.12% STTD P.

**Table 5.** Ingredient composition of Exp. 2 diets (as-fed basis)^a^

	Phase 1					Phase 2					Phase 3
*TM-SBM replacement of SBM, %* ^b^:	0	25	50	75	100	0	12.5	25	37.5	50	Common
*Calculated analysis*											
*SID AA, %*											
* Lys, %*	1.30	1.30	1.30	1.30	1.30	1.30	1.30	1.30	1.30	1.30	1.25
* Ile: Lys*	56	57	58	58	59	55	55	55	56	56	55
* Leu:Lys*	117	117	118	118	119	112	112	113	113	113	111
* Met:Lys*	36	36	36	36	36	37	37	37	37	37	40
* Met and Cys:Lys*	59	59	59	59	59	58	58	58	58	58	58
* Thr:Lys*	65	65	65	65	65	65	65	65	65	65	66
* Trp:Lys*	20	20	20	20	20	20	20	20	20	20	20.0
* Val:Lys*	70	70	70	70	70	70	70	70	70	70	73
* His:Lys*	36	35	35	35	34	35	34	34	34	34	33
*NE, kcal/kg*	2,557	2,566	2,573	2,582	2,590	2,496	2,500	2,504	2,509	2,513	2,277
*SID Lys:NE, g/Mcal*	5.08	5.07	5.05	5.04	5.02	5.21	5.20	5.19	5.18	5.17	5.49
*Lactose, %*	12.0	12.0	12.0	12.0	12.0	7.2	7.2	7.2	7.2	7.2	—
*SID Lys:CP, %*	6.46	6.53	6.60	6.68	6.75	6.55	6.60	6.64	6.68	6.72	6.29
*CP, %*	20.1	19.9	19.7	19.5	19.2	19.8	19.7	19.6	19.5	19.3	19.8
*Ca, %*	0.65	0.64	0.63	0.62	0.61	0.70	0.70	0.69	0.69	0.68	0.63
*STTD P, %*	0.44	0.44	0.44	0.44	0.44	0.38	0.38	0.38	0.38	0.38	0.29
*Ca:P*	1.00	1.00	1.00	1.00	1.00	1.15	1.15	1.15	1.15	1.15	1.04

a Phase 1 diets were fed from d 0 to 7 (2.7 kg feed budget). Phase 2 diets were fed from d 7 to 21 (4.5 kg feed budget). Phase 3 was fed to pigs from d 21 to 28.

b TM-SBM; thermo-mechanically processed soybean meal; Provisoy (Cargill Animal Nutrition, Minneapolis, MN).

c Empirical Phytase (ADM, Chicago, IL) included at 3,132 FTU/kg provided an estimated release of 0.12% STTD P.

All diets were fed in meal form and were manufactured at the New Vision CO-OP (Worthington, Minnesota). Pig weights and feed disappearance were measured on d 0, 7, 14, 21, and 28 to determine ADG, ADFI, and G:F. Fecal samples were also collected at the end of phases 1 (d 7) and 2 (d 21) from three randomly selected pigs per pen to determine percentage DM. Fecal samples were processed and analyzed using the same procedure as Exp. 1. In addition, the feces that were collected for fecal DM were also scored on a 0 to 4 scoring system: 0 = hard, pellet-like lumps; 1 = firm, formed feces; 2 = normal feces; 3 = mild looseness; and 4 = diarrhea by two observers.

### Statistical analysis

#### Experiment 1

Data were analyzed using a linear mixed model as a generalized randomized block design using the lmer function from the lme4 package in R Studio (Version 4.3.1, R Core Team. Vienna, Austria) with pen serving as the experimental unit and dietary treatment, weight block, and the associated interaction as fixed effects. Barn was included in the model as a random effect. Linear and quadratic contrasts were tested within increasing levels of SPC, excluding the ESBM treatment. The effect of 8.5% ESBM was tested by a pairwise comparison with 8.5% SPC (50% replacement of SBM). Fecal DM samples were analyzed using the fixed effects of day, treatment, block, and the associated interactions accounting for repeated measures over time.

#### Experiment 2

Data were analyzed as a randomized complete block design with a one-way ANOVA treatment structure using the lmer function from the lme4 package in R Studio (Version 4.3.1, R Core Team. Vienna, Austria). Pen was considered the experimental unit, dietary treatment was modeled as a fixed effect, and weight block was included as a random intercept. Linear and quadratic contrasts were used to test for increasing levels of TM-SBM. Fecal DM samples were analyzed using the fixed effects of day, treatment, and the associated interactions accounting for repeated measures over time. Fecal scores were summarized using the FREQ procedure of SAS OnDemand for Academics (SAS Institute, Inc., Cary, NC) and reported as a percentage of observations within each score category by treatment. Fecal score data were analyzed as a categorical variable using the GLIMMIX procedure of SAS. Results for both experiments were considered significant with *P* ≤ 0.05 and were considered marginally significant with 0.05 < *P* ≤ 0.10.

## Results

### Experiment 1

There were no significant interactions of treatment and BW block, therefore interpretation is focused on the main effect of treatment. From d 0 to 9 (phase 1), a tendency for an improvement in ADG (quadratic, *P* = 0.077) was observed as SPC replaced up to 50% of SBM resulting in a tendency for an increase (quadratic, *P* = 0.052) in d 9 BW (Table 5). Additionally, as SPC increased, G:F increased (quadratic, *P* = 0.029) with the greatest improvement at 50% SBM SID Lys replacement. When comparing 50% SPC replacement (8.5% inclusion) to 8.5% ESBM, there was a tendency for improvement in G: F for pigs fed SPC (*P* = 0.065). From d 9 to 23 (phase 2), ADG tended to increase (quadratic, *P* = 0.072) and d 23 BW increased (quadratic, *P* = 0.030) with increasing SPC, with the greatest improvement observed at 50% replacement of SBM in the diet. Additionally, as SPC increased up to 75% SBM replacement, ADFI increased (linear, *P* = 0.024). This resulted in decreased G:F as SPC replacement increased up to 100% of the SBM in the diet (linear, *P* = 0.003). There were no treatment differences between 50% SPC replacement (8.5% inclusion) and 8.5% ESBM. For the experimental period (d 0 to 23), ADFI increased (linear, *P* = 0.019) as SPC increased. Additionally, ADG and G:F increased (quadratic, *P* ≤ 0.030) with the maximum response at 50% replacement of SBM. In the common period (phase 3), no differences in ADG, ADFI, or G:F were observed due to the inclusion of SPC in the previous diets. However, d 37 BW increased (quadratic, *P* = 0.035) as SPC previously increased up to a replacement of 50% SBM. No treatment differences were observed between 50% SPC replacement (8.5% inclusion) and 8.5% ESBM.

**Table 6.** Effect of soy protein concentrate (SPC) replacement of soybean meal (SBM) on growth performance and fecal dry matter (DM) of nursery pigs (Exp. 1)^a^^,b^

	SPC replacement of SBM, %					ESBM, %^c^		*P* =		
	0	25	50	75	100	8.50	SEM	Linear^d^	Quadratic^d^	Protein Source^e^
*BW, kg*										
* d 0*	5.6	5.6	5.6	5.6	5.6	5.6	0.02	0.417	0.568	0.559
* d 9*	6.6	6.8	6.8	6.8	6.8	6.7	0.07	0.078	0.052	0.143
* d 23*	12.4	12.8	12.9	12.8	12.6	12.7	0.22	0.546	0.030	0.529
* d 37*	20.3	20.4	20.7	20.5	20.0	20.7	0.34	0.454	0.035	0.968
*Phase 1 (d 0 to 9)*										
* ADG, g*	111	134	135	133	134	121	7.1	0.055	0.077	0.176
* ADFI, g*	141	149	143	161	154	140	8.0	0.158	0.866	0.842
* G:F, g/kg*	793	907	974	839	878	862	41.9	0.451	0.029	0.065
*Phase 2 (d 9 to 23)*										
* ADG, g*	412	424	432	426	413	432	14.7	0.936	0.072	0.990
* ADFI, g*	533	560	559	572	568	551	16.7	0.024	0.254	0.620
* G:F, g/kg*	772	760	774	745	727	785	10.9	0.003	0.199	0.467
*Experimental period (d 0 to 23)*										
* ADG, g*	293	311	316	310	304	311	9.5	0.365	0.030	0.601
* ADFI, g*	378	399	396	409	406	391	11.5	0.019	0.300	0.647
* G:F, g/kg*	775	781	798	758	748	796	10.1	0.018	0.024	0.893
*Common period (d 23 to 37)*										
* ADG, g*	553	547	560	551	530	568	11.2	0.180	0.173	0.582
* ADFI, g*	815	814	831	818	788	825	25.2	0.270	0.106	0.765
* G:F, g/kg*	679	673	675	674	671	689	12.7	0.556	0.894	0.251
*Overall (d 0 to 37)*										
* ADG, g*	389	400	408	401	389	407	8.6	0.960	0.014	0.881
* ADFI, g*	539	556	561	563	550	553	15.3	0.309	0.080	0.573
* G:F, g/kg*	722	721	729	712	706	736	8.3	0.087	0.225	0.506
*Fecal DM^6^, %*										
* d 9*	22.7	23.5	24.8	25.6	24.5	21.7	1.36	0.035	0.177	0.011
* d 23*	23.9	23.2	25.1	24.8	25.2	23.3	1.36	0.128	0.999	0.145

a A total of 360 barrows (initially 5.6 ± 0.02 kg) were used with five pigs per pen and 12 replicates per treatment.

b No meaningful interactions of treatment and BW block (*P* > 0.10).

c ESBM; enzymatically treated soybean meal; HP 300 (Hamlet Protein, Findlay, OH).

d Comparing the main effects of SPC replacement of SBM excluding ESBM.

e Comparing 8.5% SPC (50% SBM replacement) and 8.5% ESBM.

6 No treatment × day interaction observed (*P* > 0.10).

Overall (d 0 to 37), increasing SPC increased ADG (quadratic, *P* = 0.014) with the greatest improvement at 50% SBM replacement and tended to decrease (linear, *P* = 0.087) G:F up to 100% SPC replacement of SBM. Additionally, ADFI tended to increase (quadratic, *P* = 0.080) up to 50% SPC replacement of SBM in the diet. There were no treatment differences between 50% SPC replacement (8.5% inclusion) and 8.5% ESBM.

Pigs fed increasing SPC had increased fecal DM on d 9 (linear, *P* = 0.035), and those fed 50% SPC replacement of SBM (8.5% inclusion) had greater (*P* = 0.011) fecal DM than pigs fed ESBM. There were no differences in fecal DM on d 23.

### Experiment 2

From d 0 to 7 (phase 1), ADG, ADFI, and d 7 BW increased (quadratic, *P* ≤ 0.003) with increasing TM-SBM up to 50% of the SBM replacement and decreased thereafter (Table 6). Additionally, G:F increased (quadratic, *P* < 0.001) as TM-SBM replacement of SBM increased up to 75% and decreased at 100% replacement of SBM. From d 7 to 21 (phase 2), ADG decreased (linear, *P* = 0.029) and G:F tended to decrease (linear, *P* = 0.072) with increasing TM-SBM. Day 21 BW increased then decreased (quadratic, *P* = 0.020) with increasing TM-SBM, which was a result of pigs being the heaviest when 25% to 50% of SBM was replaced in phase 1 followed by 12.5% to 25% replacement in phase 2. From d 0 to 21 (experimental period), ADG and ADFI increased then decreased (quadratic, *P* ≤ 0.038) with the greatest gain and feed intake observed when TM-SBM replaced 25% to 50% of the SBM in phase 1 and 12.5% to 25% of the SBM in phases 2. Gain-to-feed ratio increased (quadratic, *P* = 0.036) when TM-SBM replaced up to 50% of SBM in phase 1 and 25% in phase 2. From d 21 to 28 (common period), no significant differences were observed in ADG, ADFI, or G:F. Overall (d 0 to 28), ADG and ADFI tended to increase up to the treatment with 25 and 12.5% SBM replacement in phases 1 and 2, respectively, with performance decreasing (quadratic, *P* ≤ 0.089) with further replacement of SBM.

**Table 7.** Effect of thermo-mechanically processed soybean meal (TM-SBM) replacement of SBM on growth performance and fecal dry matter (DM) of nursery pigs (Exp. 2)^a^

	TM-SBM replacement of SBM, %							
*Phase 1:*	0	25	50	75	100		*P* =	
*Phase 2:*	0	12.5	25	37.5	50	SEM	Linear	Quadratic
*BW, kg*								
* d 0*	5.6	5.6	5.6	5.6	5.6	0.10	0.791	0.840
* d 7*	6.1	6.3	6.5	6.4	6.1	0.13	0.533	0.001
* d 21*	10.7	11.0	11.0	10.7	10.4	0.32	0.071	0.020
* d 28*	12.9	13.5	13.2	13.0	12.6	0.51	0.165	0.051
*Phase 1 (d 0 to 7)*								
* ADG, g*	57	88	103	100	65	9.9	0.205	< 0.001
* ADFI, g*	197	205	208	201	182	8.8	0.062	0.003
* G:F, g/kg*	260	431	490	495	345	43.5	0.025	< 0.001
*Phase 2 (d 7 to 21)*								
* ADG, g*	318	324	315	299	298	17.9	0.029	0.528
* ADFI, g*	422	439	429	415	410	17.1	0.111	0.162
* G:F, g/kg*	754	732	731	711	726	19.8	0.072	0.288
*Experimental period (d 0 to 21)*								
* ADG, g*	222	237	238	226	213	9.7	0.185	0.011
* ADFI, g*	339	353	348	337	327	10.4	0.069	0.038
* G:F, g/kg*	655	669	682	666	652	12.8	0.753	0.036
*Common period (d 21 to 28)*								
* ADG, g*	312	349	311	315	320	34.3	0.770	0.736
* ADFI, g*	570	624	581	578	577	37.3	0.692	0.446
* G:F, g/kg*	533	548	517	518	545	32.0	0.925	0.422
*Overall (d 0 to 28)*								
* ADG, g*	243	263	255	247	239	14.0	0.323	0.059
* ADFI, g*	393	416	403	393	388	14.5	0.205	0.089
* G:F, g/kg*	617	628	628	618	614	16.1	0.632	0.276
*Fecal DM^b^, %*								
* d 7*	11.5	21.4	16.1	18.3	17.8	1.31	0.014	0.004
* d 21*	19.0	19.5	18.9	19.0	19.8	1.31	0.774	0.786
*Removals, %*	7.2	6.8	4.4	4.4	2.8	1.63	0.014	0.759
*Mortality, %*	0.0	0.4	0.4	0.4	0.0	0.4	0.999	0.996
*Total removals and mortality, %*	7.2	7.2	4.8	4.8	2.8	1.63	0.014	0.547

a A total of 1,254 mixed-sex pigs (initially 5.6 ± 0.10 kg) were used with 18 to 20 pigs per pen and 12 pens per treatment.

b Treatment × day interaction (quadratic, *P* = 0.024).

**Figure 1:**
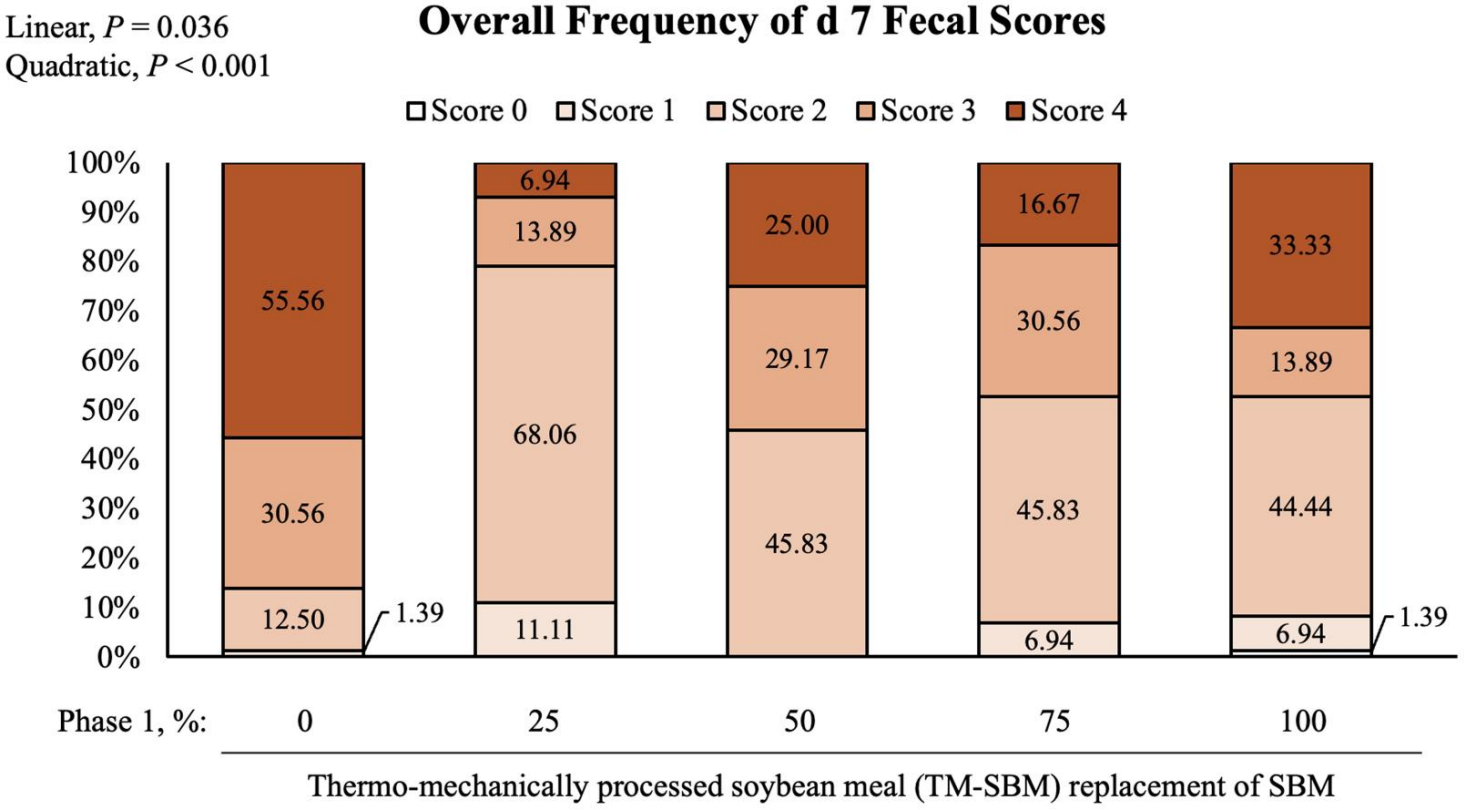
Day 7 fecal scores from exp. 2 are presented on a 4-point scale: 0 = hard, pellet-like lumps; 1 = firm, formed feces; 2 = normal feces; 3 = mild looseness; and 4 = diarrhea scored by two observers on d 7 and 21. Treatment × day interaction (quadratic, *P* = 0.017). main effect of treatment (linear, *P* = 0.041; quadratic, *P* = 0.014) and day (*P* = 0.004).

**Figure 2:**
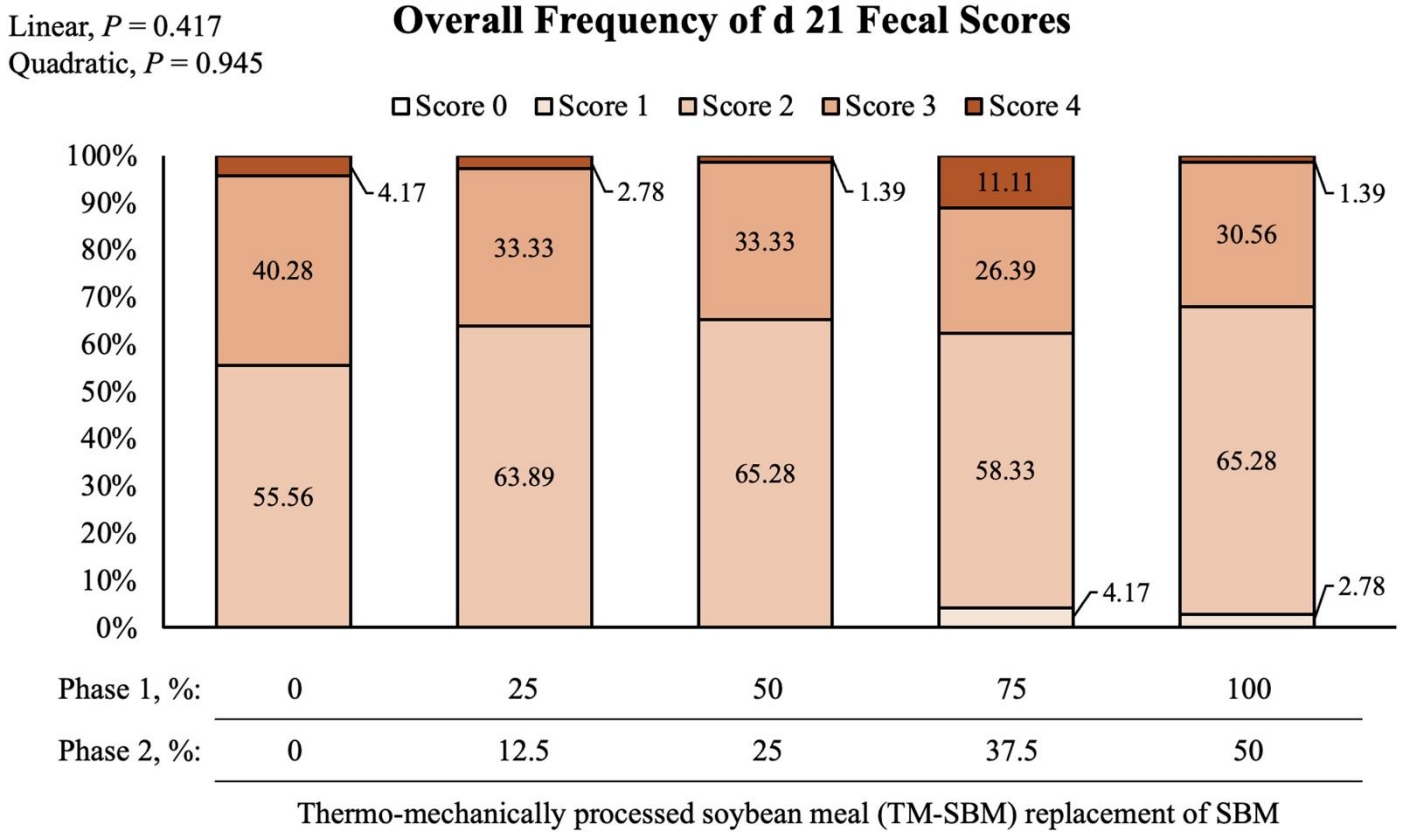
Day 21 fecal scores from exp. 2 are presented on a 4-point scale: 0 = hard, pellet-like lumps; 1 = firm, formed feces; 2 = normal feces; 3 = mild looseness; and 4 = diarrhea scored by two observers on d 7 and 21. Treatment × day interaction (quadratic, *P* = 0.017). main effect of treatment (linear, *P* = 0.041; quadratic, *P* = 0.014) and day (*P* = 0.004).

A significant treatment × day interaction was observed for fecal DM (quadratic, *P* = 0.024) driven by a treatment effect on d 7 but no treatment differences observed on d 21. On d 7, fecal DM increased as TM-SBM increased from 0% to 25% replacement of SBM (quadratic, *P* = 0.004), but then decreased at the 50% replacement level and remaining relatively consistent thereafter. Similar to fecal DM, a treatment × day interaction was observed for fecal scoring (quadratic, *P* = 0.017) driven by a treatment effect on d 7 (quadratic, *P* < 0.001) with the greatest reduction in diarrhea incidence occurring when pigs were fed diets with TM-SBM replacing 25% of the SBM (Fig. 1); however, no treatment difference in fecal score was observed on d 21 (Fig. 2).

Overall, there were no observed treatment differences in mortality; however, the percentage of removals, as well as the combined total of removals and mortality, decreased (linear, *P* ≤ 0.014) as the inclusion of TM-SBM increased from d 0 to 21.

## Discussion

Dehulled SBM is a common protein source, containing approximately 47.7% CP (NRC, 2012). Additional processing can enhance SBM’s CP content while reducing ANF that may decrease weanling pig growth. Two methods of further processing SBM include enzymatic treatment (EBSM) and thermo-mechanical processing (TM-SBM). Enzyme treatment of SBM involves the use of a specialized blend of enzymes (proteases, carbohydrases, and sometimes phytases) to break down carbohydrates and reduce ANF (Cervantes-Pahm and Stein, 2010). Alternatively, thermo-mechanically processed SBM is a method used to enhance the nutritional quality of SBM by applying heat, pressure, and shear force. The heat treatment denatures ANF like trypsin inhibitors and allergenic proteins, which can improve digestibility and nutrient absorption (Ton Nu et al. 2020). In contrast, soy protein concentrate is obtained by extracting water-soluble carbohydrates from defatted soy flakes using aqueous ethanol resulting in a product that contains a minimum of 65% CP (Wang et al. 2004).

Lenehan et al. (2007) observed that pigs fed diets containing a soy protein concentrate product included at 14.3% exhibited greater ADG compared to those fed 28.6% where SBM was completely replaced, suggesting that a high inclusion of specialty soy protein can negatively affect growth performance. This was also observed in both of the current experiments where total replacement of SBM with specialty soy products led to a reduction in growth performance relative to lower replacement levels. In a second study, Lenehan et al. (2007) observed that as soy protein concentrate increased and replaced SBM, ADG increased up to 14.3% SPC (50% replacement of SBM). Also, ADFI increased quadratically as soy protein concentrate inclusion increased to 21.4% (75% replacement of SBM). Similar results were observed in Exp. 1, where ADG increased with SPC inclusion up to 50% SBM replacement (8.5% inclusion) before decreasing with further replacement, while ADFI tended to follow a quadratic increase up to 75% SBM replacement. Likewise, this response was observed in Exp. 2, where ADG exhibited a quadratic trend, peaking at 50% and 25% TM-SBM replacement for SBM in phases 1 and 2, respectively. However, in contrast to Lenehan et al. (2007) and Exp. 1, ADFI was highest at 25% replacement in phase 1 and 12.5% in phase 2 and decreased with further replacement of SBM with TM-SBM. Overall, these data suggest that 100% replacement of SBM in nursery diets with specialty soy products under SID Lys-equivalent formulation can negatively affect growth performance, perhaps by the reduced CP, nitrogen supply, and potential limitation in NEAA availability.

The poorer performance of pigs fed the control diet in both of the current experiments may partially be attributed to hypersensitivity to SBM, although differences in feed intake and dietary protein characteristics may also have contributed. Glycinin and β-conglycinin are two major storage proteins in SBM, which account for approximately 65% to 80% of the total protein content (Thrane et al. 2017). These proteins have been shown to induce transient hypersensitivity in weaned pigs, leading to cellular-level abnormalities in the gastrointestinal tract, such as reduced villous height and crypt hypertrophy, which can negatively affect growth performance (Chen et al. 2011). This response may also increase susceptibility to disease challenges and reduce overall resiliency during the post-weaning period. In commercial nursery diets, phase 1 SBM levels are commonly limited to approximately 18% to 20%, which puts the 25 to 50% replacement levels evaluated in this study within or near practical industry application where partial reduction of SBM-associated hypersensitivity may be most beneficial. In Exp. 2, pigs fed the control diet with no TM-SBM not only had reduced feed intake but also had a higher percentage of pigs removed from the study due to poor growth and losing weight, suggesting compromised gut health associated with SBM sensitivity.

Lenehan et al. (2007) investigated the palatability of soy protein concentrate in nursery pig diets and observed a preference for a diet containing 40% SBM over a diet where soy protein concentrate completely replaced SBM (28.6% inclusion). The product used by Lenehan et al. (2007) was an aqueous alcohol-extracted SPC that undergoes moist extrusion, which may have contributed to the differences in palatability. Additionally, Solà-Oriol et al. (2011) observed that pigs preferred soy protein concentrate when added at 20% of the diet compared to a control diet containing 20% ESBM. However, in Exp. 1, no differences in feed intake were observed between pigs fed SPC and those fed ESBM. While high SPC replacement of SBM did not appear to affect feed intake during the experimental period in Exp. 1, fully replacing SBM with 17% SPC was associated with decreased growth performance.

A possible explanation for the reduction in growth performance at 100% replacement of SBM with SPC or TM-SBM may be a deficiency of non-essential amino acids (NEAA) due to the higher SID Lys to CP ratio or lower CP level when SPC or TM-SBM completely replaced SBM in the diet. The NRC (2012) estimates a minimum of 3.63 and 3.29% total dietary N for pigs weighing 5 to 7 kg and 7 to 11 kg, respectively, along with SID Lys levels of 1.50% and 1.35%, respectively. Based on these values, the maximum SID Lys:N ratio can be estimated, or total N can be converted to a CP basis using a factor of 6.25 to obtain the SID Lys:CP ratio. Based on NRC (2012) estimates, diets should be formulated to maintain a SID Lys:CP ratio below 6.61% for 5- to 7-kg pigs and 6.57% for 7 to 11 kg pigs. In both experiments, the diets with 100% SBM replacement resulted in SID Lys:CP ratios that exceeded the estimate (6.93 and 7.00% in phases 1 and 2, respectively, for Exp. 1 and 6.75 and 6.72% in phases 1 and 2, respectively, for Exp. 2). This is because of the greater SID Lys:CP ratio of the specialty protein products compared to SBM. Previous research (Smallfield et al. 2025a, 2025b) suggests that G:F decreases when the SID Lys: CP ratio exceeds the estimated SID Lys:CP ratio, which was observed in the studies herein. Therefore, reduced performance at complete replacement cannot be attributed solely to the specialty protein source itself.

Numerous studies have evaluated the effects of enzymatically treated SBM as a partial replacement for SBM in nursery pig diets. Jones (2017) fed diets ranging from 6.67 to 20.00% ESBM in phase 1 and 5.00 to 15.00% in phase 2 and observed that pigs fed the lowest inclusion (6.67 and 5.00% in phases 1 and 2, respectively) had improved growth performance during the experimental period compared to pigs fed the higher levels. The reduction in ADG was primarily driven by decreased ADFI, possibly due to palatability issues, as feed intake decreased with increasing enzymatically treated SBM. The bitter taste associated with high enzymatically treated SBM additions is thought to result from proteolytic enzymes exposing the hydrophobic AA located within the interior of the protein (Liu et al. 2022).

The ideal level of enzymatically treated SBM in weanling pig diets varies across studies. Tan et al. (2024) found 4% enzymatically treated SBM to be optimal compared to either 2 or 8%. Ruckman et al. (2020) reported 7% ESBM as the ideal inclusion for growth compared to 14 or 21%. However, the enzymatically treated SBM used by Tan et al. (2024) was different than the source used in our study. Because the previous research suggested that high amounts of enzymatically treated SBM can decrease growth performance, 8.5% ESBM was used to compare with 8.5% SPC (50% SBM replacement) in Exp. 1. Yang et al. (2007) compared pigs fed nursery diets containing 8% soy protein concentrate and 8% ESBM and reported that pigs fed soy protein concentrate had greater ADG than those fed ESBM during the first two weeks post-weaning. This observed advantage in growth performance with SPC inclusion contrasts with the results observed in Exp. 1 where there were no treatment differences observed between pigs fed diets containing 8.5% SPC and those fed 8.5% ESBM. However, this may be due to Yang et al. (2007) using a soy protein concentrate source other than the one used in the current study.

The linear increase in fecal DM with increasing SPC and TM-SBM during the first phase may be associated with the decreasing CP content of the diet as SPC and TM-SBM increased. A low CP diet reduces the availability of substrate for bacteria fermentation, thereby improving fecal consistency (Pieper et al. 2016). As pigs age and their digestive tract matures, they become less susceptible to the effects of SBM hypersensitivity, intestinal barrier dysfunction, and pathogenic growth (Han et al. 2024). This likely explains the lack of treatment differences in fecal DM in both experiments at the end of phase 2. Fecal DM was lowest with pigs fed the control diet with no specialty soy protein in both experiments, which may partially reflect the greater concentration of glycinin, β-conglycinin, and trypsin inhibitor in SBM. These anti-nutritional factors may contribute to digestive disturbances during early weaning (Li et al. 1991). Overall values for fecal DM were lower in Exp. 2 than that observed in Exp. 1. This might be explained by environment where Exp. 2 was conducted in a commercial research facility while Exp. 1 took place in a university setting. Montagne et al. (2012) observed that pigs raised in an environment with greater microbial pressure had lower fecal DM compared to those in a cleaner environment. Because intestinal morphology, permeability, or inflammatory markers were not measured, fecal DM should be interpreted with caution and not necessarily a direct measure of gut health.

Fecal scores on d 7 exhibited a quadratic response with increasing TM-SBM, where pigs fed the control diet and those fed the highest level of TM-SBM had the greatest frequency of diarrhea. Fecal scores are commonly used in the industry as they provide a simple method for assessing the incidence of diarrhea, though they can be more subjective compared to measuring fecal DM. However, these findings suggest that fecal DM measurements and fecal scoring provided consistent trends.

Although there are a few studies evaluating the effects on fecal consistency when replacing SBM with SPC, more research has compared SPC to other protein sources. Deng et al. (2022) found no differences in fecal consistency across varying levels of soy protein concentrate replacement of animal protein supplements (0, 33, 66, and 100%), despite observing differences in growth performance. Similarly, Wen et al. (2018) reported no difference in fecal scores between pigs fed a soy protein concentrate with 19% CP and soy protein concentrate with 23% CP. This finding is particularly interesting, given that previous research suggests high CP diets can increase the incidence of diarrhea (Limbach et al. 2021). However, Wen et al. (2018) observed that pigs fed soy protein concentrates had less diarrhea than those fed a control diet formulated to 17% CP. In contrast, Ma et al. (2019) observed that pigs fed a SBM-based control diet without processed soy products had a greater incidence of diarrhea than those fed 7.51% soy protein concentrate during the first two weeks post-weaning. This was similarly observed in both experiments where the diets with no specialty soy products had the lowest fecal DM after phase 1.

From a practical standpoint, partial replacement of SBM with SPC or TM-SBM may be most applicable during the first 2 to 3 weeks post-weaning when specialty protein inclusion is highest and digestive challenges are greatest. However, ingredient cost and overall diet formulation constraints should also be considered. In summary, these data suggest that replacing SBM with 25 to 50% SPC or TM-SBM in phase 1 diets improves nursery pig growth performance and fecal DM. Furthermore, 50% SPC replacement of SBM (8.5% inclusion) resulted in similar growth performance to ESBM, while improving fecal DM. Fecal scoring results align with trends observed in fecal DM, suggesting that either method can be utilized. Overall, complete replacement of SBM with specialty soy products under the dietary conditions used in these experiments was associated with poorer growth performance, suggesting that successful replacement may depend on maintaining adequate total nitrogen supply and amino acid balance.

## List of abbreviations

AA: amino acids
ADG: average daily gain
ADFI: average daily feed intake
ANF: anti-nutritional factors
BW: body weight
CP: crude protein
DM: dry matter
ESBM: enzymatically treated soybean meal
G: F: gain-to-feed
NE: net energy
NEAA: non-essential amino acids
SBM: soybean meal
SID: standardized ileal digestible
SID Lys: CP: standardized ileal digestible lysine to crude protein
SID Lys: N: standardized ileal digestible lysine to nitrogen
SPC: soy protein concentrate
TM-SBM: thermo-mechanically processed soybean meal
